# Effect of short-term high-temperatures on the growth, development and reproduction in the fruit fly, *Bactrocera tau* (Diptera: Tephritidae)

**DOI:** 10.1038/s41598-020-63502-w

**Published:** 2020-04-14

**Authors:** Yuyu Huang, Xiangpeng Gu, Xiaoqin Peng, Mei Tao, Guohua Chen, Xiaoming Zhang

**Affiliations:** College of Plant Protection, Yunnan Agricultural University, National Key Laboratory for Conservation and Utilization of Biological Resources in Yunnan, Kunming, 650201 China

**Keywords:** Plant sciences, Zoology

## Abstract

*Bactrocera tau* (Walker) (Diptera: Tephritidae) is an economically important invasive pest, that is capable of seriously reducing the quality and yield of vegetables and fruits, it was first recorded from Fujian province in 1849 and later introduced to Yunnan province in 1912 as a result in trade fruits and vegetables of China. In recent years, with the onset of global climate change and the accompanying increase in the greenhouse effect, elevated climatic temperatures have become one of the main environmental factors affecting growth and reproduction in insects, and the optimal developmental temperature of *B. tau* was found to be from 25 °C to 31 °C, the growth, development and reproduction of *B. tau* are normal under the optimal temperature conditions. In order to determine the repercussions that elevated temperature have on *B. tau*, we assessed the effects that short-term (12 h) high-temperature exposures (34 °C, 36 °C, 38 °C, 40 °C, 42 °C, 44 °C, 46 °C, and 48 °C) had on the growth, development and reproduction of *B. tau* at different developmental stages of the fly. The results showed that the survival rate of *B. tau* gradually decreased in all stages following exposure to short-term high-temperatures. The pupal stage was the least sensitive to increased temperatures. The pupae withstood the highest lethal temperature, having an LT_50_ of 42.060 °C, followed by female adults (40.447 °C), male adults (40.013 °C), and larvae (36.740 °C). The egg stage, which was the most susceptible to heat increases, had the lowest LT_50_ (38.310 °C). No significant effects were observed in the developmental stages of *B. tau* at temperatures from 24 °C to 38 °C. The development duration was significantly prolonged at 40 °C (*P* < 0.05) in the eggs (2.830d), larvae (7.330d), and pupae (8.170d) (*P* < 0.05). *B. tau* was unable to survive at temperatures above 42 °C. The pre-oviposition of female adults was extended, the average egg number per female showed a downward trend, the longevity of adults gradually shortened, and the ratio of female to male offspring increased as temperature increments were increased. In summary, short-term high-temperatures over 42 °C were not suitable for successful development of *B. tau*, while short-term high-temperatures over 40 °C were not suitable for successful reproduction in *B. tau*.

## Introduction

The fruit fly *Bactrocera tau* (Walker) (Diptera: Tephritidae) is a major economic pest on cucurbitaceous plants, tomatoes, and other fleshy fruits^1^. *B. tau* was first recorded from China from Fujian province in 1849 by Walker^2^. The species was subsequently discovered in Yunnan, Guangdong, and Sichuan provinces from 1912 to 1913^3–5^, and has since dispersed rapidly through much of southern China. From 2000 to 2004, *B. tau* has been reported from much of south Asia, southeastern Asia as well as the Solomon Islands^6–9^. In these countries, the *B. tau* has severely reduced quality and yield of vegetables and fruits. The *B. tau* has caused 21–34% and 21–32% yield losses of *Siraitia grosvenorii* and *Cucurbita moschata* respectively in Taiwan^10^, and approximately 5.6% infestations of ripen *Luffa acutangula* in Thailand have been damaged by *B. tau*^11^, etc. The larvae cause the bulk of the damage by their feeding inside of the host plants, while the female adults who unsuccessfully lays eggs are responsible for secondary damage to the plants by physically puncturing. Feeding by the larvae causes the pulp to rot, making the fruit unsuitable for human consumption and almost worthless commercially. Because *B. tau* has been very adaptable to changes in its environment, maintaining its ability to feed and oviposit in many diverse situations, it is regarded as a major economic pest and included on the quarantine lists of many countries and regions^12–14^.

Average global temperatures have risen for the past several decades, accompanying the documented changes in the world’s climate^15^. The range, frequency, and duration of higher temperatures have continued to increase under the climatatic warming trend^16–18^. This is demonstrable in several regions of southern China where the maximum daily temperatures during summer often reaches 40 °C or higher for an extended period of several hours each day, compared to the daily high-temperatures in previous decades. It also appears that the daily high-temperatures are continuing to increase in these areas^19,20^. Because insects are typical ectothermic organisms they are significantly affected by changes in their ambient temperature^21,22^. Higher temperatures may have either positive or negative effects on oviposition^23^, mating^24^, and thermostatic behavior^25^. Insects are typically unable to recover from thermal damage because they lack sufficient time or adequate resources when affected by extreme heat^26^. Extreme heat may also affect the reproductive, nervous, endocrine, and immune systems as well as biomacromolecular synthesis in insects^27,28^. The neurally-based clock and development of *Sarcophaga crassipalpis* (Macquart) (Diptera: Sarcophagidae) were in effect broken and retarded by being subjected to heat shock and to cold shock^29^. The normal development of reproductive organs were inhibited by high-temperature treatment (≥ 46 °C) in *Spodoptera exigua* (Hübner) (Lepidoptera: Noctuidae)^30^; the reproductive ability and offspring survival rate declined in *Trialeurodes vaporariorum* (Westwood) (Hemiptera: Aleyrodidae)^31^ and declined in *Bemisia tabaci* (Gennadius) (Hemiptera: Aleyrodidae)^32^ after short-term heat treatment (≥ 37 °C); the survival rate, longevity, and reproduction of adult *Myzus persicae* (Sulzer) (Hemiptera: Aphididae) decreased after exposure to short-term high-temperatures (≥ 40 °C)^33^. Liu’s^34^ study found that high-temperature significantly effected the behavior, growth, survival, and mating of *B. tau*. under laboratory conditions, the optimal developmental temperature of *B. tau* was found to be from 25 °C to 31 °C. When the ambient temperature exceeded 34 °C, the pupation rate was extremely low, and the adult emergence rate of those that did succeed in pupating was also exceptionally low. These temperatures were also not suitable for the growth and reproduction of *B. tau*^34^. It is inevitable for *B. tau* to experience short-term heat stress during the summer in the southern regions of China. Our previous study only focused on rapid death caused by exposure to long-term high-temperature; however, short-term high-temperature can also have major detrimental effects on growth, development and reproduction. In effect, insects may slowly die after being subjected to short-term high-temperature^21^. Thus, the heat stress effect can potentially cause a sustainable impact on adult insects^35,36^, which may even be carried over into the next generation^37^. The present study was conducted in response to the progressively more extreme summer temperatures that are becoming commonplace in southern China, to investigate the effects of the warming environment on the development of the eggs, larvae, and pupae of *B. tau*, and then to determine how disruptive the expected increases in environmental temperatures will be on the life history of the fly, including its reproduction, survival, and potential changes in the sex ratio of adults. It is also hoped that the information obtained in the study will provide a scientific basis for further exploration of the temperature adaptation mechanism and eventually to help in formulating a comprehensive control program for *B. tau*.

## Materials and methods

### Insect rearing

*B. tau* were initially collected from a pumpkin field (*Cucurbita moschata* Duchesne) in Mengzi City (103°23′E, 23°23′N), Yunnan Province, south China. A stable experimental population of *B. tau* was established in our lab and cultured under constant conditions in an environmental chamber set at 24 ± 1  °C; 14: 10 h (light: dark) photoperiod, and 50 ± 5% RH. The *B. tau* culture has been reared for more than five generations. Adult *B. tau* were fed on yeast extract (Beijing Aoboxing, China) and water. Larvae were fed on artificial diet consisting of wheat bran, yeast powder, sugar, agar powder, water, and other microelements^38^.

### Variable thermal treatments and materials used

Short-term high-temperature treatments were set at a range of temperature increments including 34 °C, 36 °C, 38 °C, 40 °C, 42 °C, 44 °C, 46 °C, 48 °C, and 24 °C (control). Eggs, larvae, pupae, and adults were placed in environmental chambers, under a 14: 10 h (light: dark) photoperiod and: 50 ± 5% RH after being exposed for 12 h in a separate environmental chamber set to one of the above temperatures, the details of the experimental treatment are as follows. Each temperature was a separate treatment and each treatment was replicated four times.

Organic marrow (*Cucurbita pepo* L.) and pumpkin (*Cucurbita moschata* Duchesne) were purchased from a Walmart supermarket and stored in a refrigerator at 4 °C.

### Effects of short-term high-temperature on egg, larval, and pupal development

Female adults oviposited in pumpkins and we get eggs from pumpkins. Eggs produced within a 24 h period were selected for each treatment and divided into groups of 10. Each group of 10 eggs were transferred using a fine bristled brush to pieces of 1 cm thick *C. pepo* in a 7.5 cm culture dish. The culture dishes containing the eggs were placed in an environmental chamber and heat-shocked at one of the above treatment temperatures for 12 h. The eggs were then transferred to a separate environmental chamber (24 °C) and allowed to hatch. The development and survival of the eggs were inspected by stereoscope and recorded every 24 hours until all eggs were either black-colored or had died (dehydrated eggs were considered to be inviable).

One-day-old larvae were selected using a special disposable paper cup, with 10 larvae comprising a group. The size of the disposable paper cup was 75 mm top × 53 mm bottom × 90 mm deep. The larvae that hatches from egg to larva within 24 hours were called one day-old larvae, the larvae were obtained from the standard colony. Larvae were feed on 4 cm slices of *C. pepo* and artificial diet in special disposable paper cups. The paper cups were placed in a clear glass jar (12 cm long × 9 cm width × 12 cm deep) and the opening covered with gauze. The clear glass jars containing the larvae and paper cups were then placed in an environmental chamber that had been set in advance at the predetermined heat shock temperature and treated for 12 h. The larvae were then placed in another environmental chamber (24 °C) and allowed to feed. Additional *C. pepo* was added as needed. After pupation, the paper cups were placed in pots (36 cm top × 28 cm bottom × 11 cm deep), containing a 3 cm deep layer of sand. The top of the paper cup was covered with three layers of gauze that was moistened by watering can to replenish moisture. The development and survival of larvae were recorded every 24 hours until pupation or death.

One-day-old pupae were selected as test materials, and each set of 10 pupae was placed in a 7.5 cm culture dish containing immersed by water filter paper. The culture dishes were placed in clear glass jars and enclosed with gauze sleeves. The jars containing the pupae were then placed in an environmental chamber at the predetermined temperature and heat-stressed for 12 h. Immediately afterwards, the pupae were removed and placed in another environmental chamber (24 °C), and then pupae will wait to emerged and do not need to be fed. Water was sprayed regularly as needed. The development and survival of the pupae were recorded every 24 hours until all pupae had emerged or died (discolored or desiccated pupae were considered to have died). Each different temperature was a treatment, and each treatment was replicated four times^38^.

### Effect of short-term high-temperature on adult survival, reproduction, longevity, and offspring ratio

One-day-old adults were selected, with each pair of females and males placed in a clear glass jar containing two 7.5 cm culture dishes used to hold water and yeast extract. Glass jars were enclosed with gauze allowing for addition of water and food as needed. Ten pairs of *B. tau* were considered as one treatment. The clear glass jars containing adult insects were placed in an environmental chamber set up in advance and heat-shocked for 12 h. After the heat shock, adults were removed and placed in separate jars containing a fresh 1 cm^3^ cube of *C. moschata* for oviposition which was changed every 24 hours. The pre-oviposition duration, the number of eggs oviposited per day, and the average egg numbers per female were recorded until the death of all adults. The eggs were collected daily and were grouped by different laying times into a cage for feeding insects, where they were placed in *C. moschata* and *C. pepo*. The offspring of *B. tau* were recorded after the adults emerged for ratio of female to male offspring.

### Data analyses

The heat resistance test data for *B. tau* was analyzed using SPSS 22.0 for One-Way ANOVA. Tukey’s multiple comparison method was used to compare significant differences among different temperatures of *B. tau* of the same stage. The bar graphs were drawn using Origin 2018.

#### Computational formula

1. The corrected survival rate was the survival rate of the treatment group corrected by the natural survival rate of the control group. The survival rate percentages in the Table 1 were calculated using computational formula of corrected survival rates.

**Table 1:** Percent survival of different development stages and sexes of *Bactrocera tau* at different temperatures.

Temperature	Development stage				
	Egg	Larva	Pupae	Male	Female
24	96.67 ± 3.33a	93.33 ± 3.33a	100.00 ± 0.00a	100.00 ± 0.00a	100.00 ± 0.00a
34	80.00 ± 5.77ab	70.00 ± 5.77ab	100.00 ± 0.00a	100.00 ± 0.00a	100.00 ± 0.00a
36	66.67 ± 6.67b	53.33 ± 8.82bc	100.00 ± 0.00a	100.00 ± 0.00a	100.00 ± 0.00a
38	56.67 ± 8.82b	46.67 ± 8.82bc	96.67 ± 3.33a	90.00 ± 5.77a	93.33 ± 3.33ab
40	53.33 ± 8.82b	30.00 ± 5.77 cd	93.33 ± 3.33a	56.67 ± 8.82b	76.67 ± 8.82b
42	10.00 ± 0.00c	13.33 ± 3.33de	70.00 ± 11.55b	6.67 ± 3.33c	6.67 ± 6.67c
44	3.33 ± 3.33c	0.00 ± 0.00e	0.00 ± 0.00c	0.00 ± 0.00c	0.00 ± 0.00c
46	6.67 ± 6.67c	0.00 ± 0.00e	0.00 ± 0.00c	0.00 ± 0.00c	0.00 ± 0.00c
48	0.00 ± 0.00c	0.00 ± 0.00e	0.00 ± 0.00c	0.00 ± 0.00c	0.00 ± 0.00c
df	8.18	8.18	8.18	8.18	8.18
F	40.93	42.63	130.98	170.48	163.58
P	0.0001	0.0001	0.0001	0.0001	0.0001

Note: Data are means ± *SE*. Different lowercase letters in each column (stage) denote significant difference in percent survival at different temperatures within that stage after analysis using Tukey’s test (*P* < 0.05). The *F* value is an indicator of ANOVA analysis, which usually compares the differences between groups. When the value of *F* is larger and the value of *P* is smaller, the result is more reliable. When *P* < 0.05, there was a significant difference between groups, when *P* > 0.05 or *P* = 0.05, there was no significant difference between groups. The letters df denote degrees of freedom.
  Corrected survival rates: Corrected survival rate (%) = (the number of viable insects in the treatment group / the number of initial insects in the test group + the number of dead insects in the control group / the total number of insects in the test)^33^.
2. LT_50_ (lethal temperature) was defined as the temperature at which half of the tested animals will die within a specified time period. The LT_50_ reflects the insect’s resistance to high or low temperatures.

Equation:

$${\rm{S}}({\rm{x}})=[{\rm{\exp }}({\rm{a}}-{\rm{bx}})]/[1+{\rm{\exp }}({\rm{a}}-{\rm{bx}})]$$

S(x) is the mortality (%) of the population at a given time or low temperature; a and b are model parameters. When the mortality “S(x)” is at 50%, i.e. a-bx = 0, x = a/b, the value of x is the lethal temperature (LT_50_)^39^.

## Results

### Survival rates of different stages exposed to different short-term high-temperatures

The corrected survival rates of different stages of *B. tau* were shown to be significantly affected by short-term high-temperature stress (at 34 °C, 36 °C, 38 °C, 40 °C, 42 °C, 44 °C, 46 °C, 48 °C and 24 °C for 12 h). The values for the different development stages were: eggs (*F*_8,18_ = 40.93, *P* = 0.0001), larvae (*F*_8,18_ = 42.63, *P* = 0.0001), pupae (*F*_8,18_ = 130.98, *P* = 0.0001), adult males (*F*_8,18_ = 170.48, *P* = 0.0001), and adult females (*F*_8,18_ = 163.58, *P* = 0.0001) (Table 1). When the treatment temperature reached 36 °C, the corrected survival rate of eggs and larvae was significantly lower than that of the control temperature, however, when the treatment temperature reached 40 °C, the corrected survival rate of adult males and females was significantly lower than that of the control temperature, the corrected survival rate of only the pupae was significantly lower than the corrected survival rate at the control temperature when the temperature reached 42 °C. The pupae were the most heat tolerant to high-temperatures with a corrected survival rate of 70.00% at 42 °C (Table 1). Adults, however, were the least tolerant of higher heat regimes, having a corrected survival rates of only 6.67% at 42 °C, and with a 0% corrected survival rate at temperatures above that, indicating that they were extremely sensitive to high-temperature stress (Temperatures > 40 °C) (Table 1). The survival rate of all stages (from eggs through adults) decreased sharply with increases in all of the tested temperatures. All of the developmental stages, with the exception of eggs, were unable to tolerate temperatures above 44 °C. The eggs were able to survive until temperature reached 46 °C (Table 1). The corrected survival rates of adult males were similar to those of the females, with both survival rates shown to decrease with increases in temperature. The heat-tolerance of adults female were slightly higher than that of males (Table 1).

### **LT**_**50**_**of different stages exposed to different short-term high-temperatures**

Pupae, with an LT_50_ of 42.06 °C, exhibited the greatest heat tolerance among all of the development stages (Table 2). The LT_50_ values for the different stages and sexes of *B. tau* were: pupae (42.06 °C) > adult females (40.447 °C) > adult males (40.013 °C) > larvae (36.74 °C) > eggs (38.31 °C) (Table 2).

**Table 2:** LT_50_ temperatures of different stages and sexes of *Bactrocera tau* after 12 hr exposure.

Stage	Hours after treatment	LT_50_ (95%FL)	Regression equation	R^2^
Egg	12	38.31 (37.433–39.104)	Y = 18.68X−29.61	0.743
Larva	12	36.74 (35.712–37.580)	Y = 17.28X−27.04	0.786
Pupae	12	42.06 (41.057–43.189)	Y = 18.71X−31.04	0.358
Male	12	40.013 (39.550–40.480)	Y = 53.75X−86.11	0.904
female	12	40.447 (40.009–40.901)	Y = 43.67X−70.47	0.714

Note: 95% fiducial limits in parentheses, LT_50_ units are in degrees celsius (°C). R^2^ is the correlation coefficient. Temperature levels are 24 °C (control), 34 °C, 36 °C, 38 °C, 40 °C, 42 °C, 44 °C, 46 °C, and 48 °C.

### **Lengths of developmental periods of different stages of*B. tau*exposed to different short-term high-temperatures**

The egg, larval, and pupal developmental periods of *B. tau* exhibited significant differences after 12 h exposure to short-term high-temperature stress (Table 3). The developmental periods of the eggs, larvae, and pupae were significant longer than those from the control temperature when the temperature was over 40 °C (Table 3). At 40 °C, the development periods of the eggs and larvae were significantly longer than those in the control insects (eggs: *F*_1,10_ = 16.2000, *P* = 0.0024; larvae: *F*_1,10_ = 22.8380, *P* = 0.0007), however, the development periods of the pupae were significantly longer than those at the control temperature when the treatment temperature reached 38 °C (*F*_1,10_ = 10.9460, *P* = 0.0079). Increases in the developmental duration of *B. tau* reflected the increases in the treatment temperatures, reaching their maximum length when stressed at 42 °C.

**Table 3:** Lengths of the egg, larval, and pupal developmental periods of *Bactrocera tau* after 12 h exposure to short-term high-temperature stress.

Temperature	Egg	Larva	Pupae
24	1.33 ± 0.21c	5.17 ± 0.31c	6.17 ± 0.31c
34	1.67 ± 0.21bc	5.33 ± 0.33c	6.67 ± 0.33bc
36	2.00 ± 0.37abc	5.83 ± 0.31bc	7.17 ± 0.31abc
38	2.33 ± 0.33abc	6.33 ± 0.49bc	7.67 ± 0.33ab
40	2.83 ± 0.31ab	7.33 ± 0.33ab	8.17 ± 0.31a
42	3.17 ± 0.31a	8.17 ± 0.48a	8.33 ± 0.33a
df	5,30	5,30	5,30
F	5.57	9.47	7.07
P	0.0010	0.0001	0.0002

Note: Data are means ± *SE*. Different lowercase letters in each column (stage) denote significant difference in developmental time at different temperatures within that stage after analysis using Tukey’s test (*P* < 0.05).

### **Preoviposition of*B. tau*females after exposure to short-term high-temperature**

**Figure 1:**
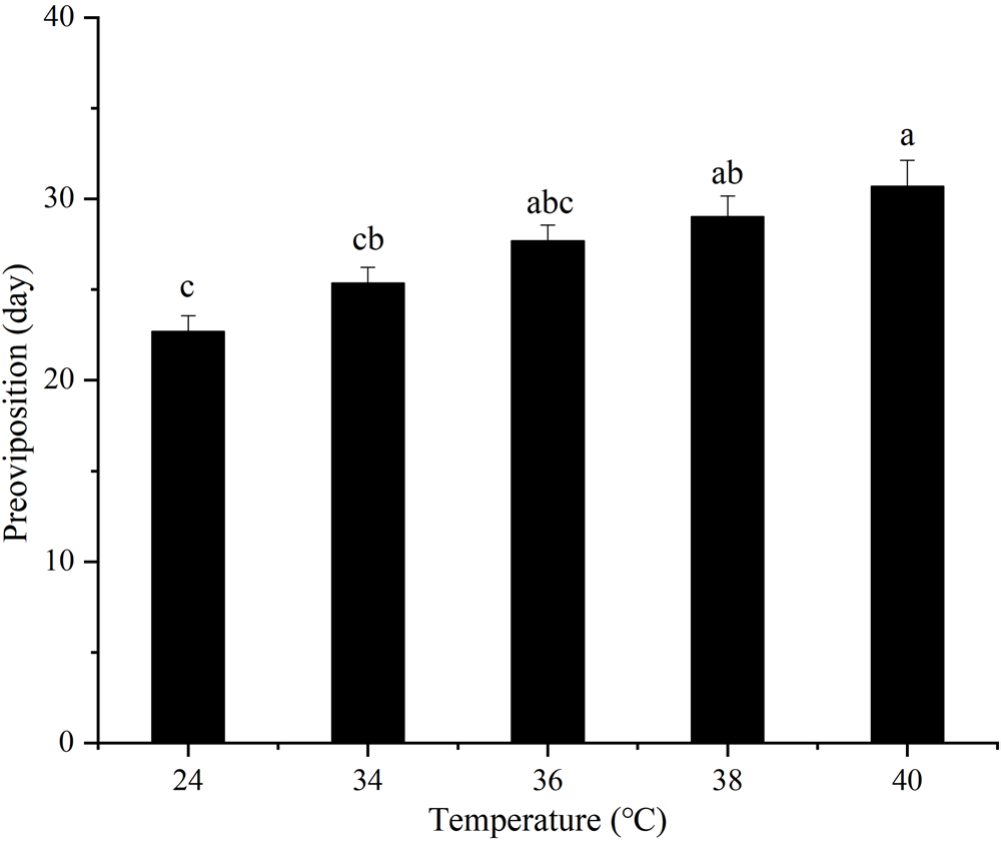
Preoviposition durations of *Bactrocera tau* (mean + *SE*) females exposed for 12 h to different short-term high-temperatures (24 (control), 34, 36, 38, and 40 °C). Different lowercase letters denote significant difference among different temperatures after analysis using Tukey’s test (*P* < 0.05). All adults had died before reaching sexual maturity after exposure to temperatures of 42 °C (or greater).

Compared to the control, the preoviposition duration was significantly effected by short-term high-temperature (*F*_4,10_ = 8.53, *P* = 0.0029). Specifically, the preoviposition time was significantly prolonged at 36 °C compare to flies at the control temperature (*F*_1,4_ = 43.0690, *P* = 0.0028) (Fig. 1). The preoviposition time period was the longest at 40 °C. Overall, the preoviposition duration gradually lengthened as temperatures increased (Fig. 1).

### **Egg production in female*B. tau*after exposure to short-term high-temperature**

**Figure 2:**
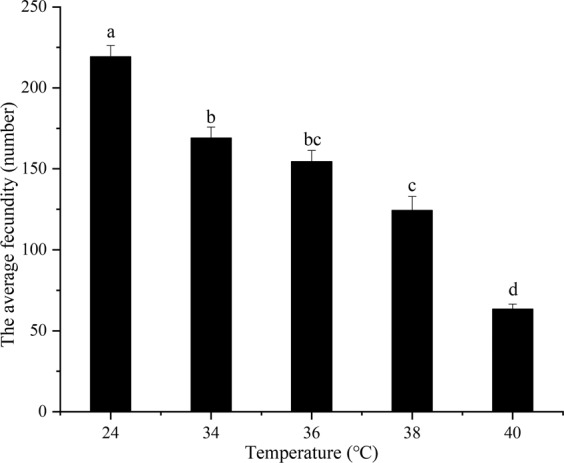
Egg production in *Bactrocera tau* (mean + *SE*) females exposed for 12 h to different short-term high-temperatures (24 (control), 34, 36, 38, and 40 °C). Different lower-case letters denote significant differences among different temperature after analysis using Tukey’s test (P < 0.05). All female adults had died before temperatures reached 42 °C.

Female egg production was significantly affected by exposed to high-temperature for 12 h (*F*_4,10_ = 73.35, *P* = 0.0001). Egg production was significantly reduced at 34 °C compared to the control temperature (*F*_1,4_ = 26.8490, *P* = 0.0066), and reached its lowest level when the treatment temperature reached 40 °C (Fig. 2). Overall, female egg production was gradually reduced as temperatures rose from 34 °C to 40 °C.

### **Logevity of*B. tau*after exposure to short-term high-temperature**

**Figure 3:**
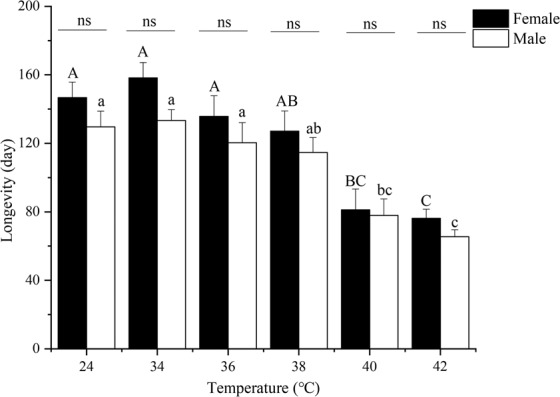
Longevity of *Bactrocera tau* (mean + *SE*) adults exposed for 12 h to different short-term high-temperatures (24 (control), 34, 36, 38, 40, and 42 °C). Different upper-case and lower-case letters denote significant differences among different temperatures in adult females and males after analysis using Tukey’s test (P < 0.05). Since all adults had died before reaching sexual maturity when temperatures reached 44 °C the 44 °C, 46 °C, and 48 °C treatments were omitted. The letters “ns” signify that there were no significant differences between adult females and males at the same temperature (P < 0.05) using Tukey’s test (P < 0.05).

The longevity of the adults was also significantly affected by short-term high-temperature exposure (Fig. 3). Adults of both sexes reached their longest longevity at 34 °C, with longevity decreasing as temperatures rose from 36 °C to 42 °C. Adult longevity was significantly higher in flies raised at the control temperature than in those raised at temperatures above 40 °C (male: *F*_1,4_ = 15.3900, *P* = 0.0172; female: *F*_1,4_ = 18.6450, *P* = 0.0125). The longevity of the adults male and female was the longest at 34 °C, however, there were no significant differences in the longevity of females and males exposed to the same temperature treatments (control: *F*_1,4_ = 1.753, *P* = 0.2561; 34 °C: *F*_1,4_ = 5.144, *P* = 0.0859; 36 °C: *F*_1,4_ = 0.820, *P* = 0.4163; 38 °C: *F*_1,4_ = 0.7180, *P* = 0.4446; 40 °C: *F*_1,4_ = 0.0430, *P* = 0.8459; 42 °C: *F*_1,4_ = 2.6180, *P* = 0.1809). Overall, the longevity of the adults male and female slightly increased when the treatment temperature went from 24 °C to 34 °C, and then gradually shortened with further increases in temperature from 34 °C to 42 °C. Adult females had a greater heat tolerance than adult males. Adult females in the control were longer lived than the males (*F*_1,4_ = 1.753, *P* = 0.2561).

### **Female to male sex ratios in*B. tau*offspring after exposure to short-term high-temperature**

**Figure 4:**
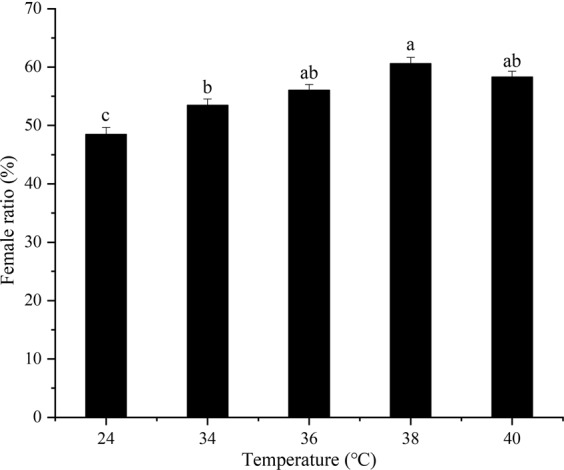
Female ratio of offspring of *B. tau* (mean + *SE*) exposed to different short-term high-temperatures (24 (control), 34, 36, 38, and 40 °C) for 12 h. Different lowercase letters denote significant differences among different temperature using Tukey’s test (*P* < 0.05). All adults had died prior to reaching sexual maturity before temperatures reached 42 °C.

There was a significant difference in the female sex ratio favoring females in the offspring of *B. tau* after short-term high-temperature treatment compared to *B. tau* in the control temperature (*F*_4,10_ = 19.25, *P* = 0.0001). The ratio of females in the offspring was highest at 38 °C and lowest at 34 °C with ratios of 60.57% (*F*_1,4_ = 56.433, *P* = 0.0017) and 53.47% (*F*_1,4_ = 9.829, *P* = 0.0350), respectively, both significantly higher than in the controls. (Fig. 4). In general, the female ratio of *B. tau* offspring gradually increased as the treatment temperature increased, reaching its highest point at 38 °C.

## Discussion

Average temperatures have been gradually rising as a result of global climatic changes, with the frequency of extreme high-temperature events affected by small increases in average temperature^17^. In some regions of China today, the maximum daily summer temperature will reach or exceed 40 °C for periods of several hours, while the average daily temperatures have continued to increase in the last few years^19,20^. The intensity and duration of temperature change can have either a negative or positive effect on the growth and metabolism of insects, which, in turn, can affect the life activities of insects within a given temperature range. Therefore, temperature plays a critical role in insect growth, reproduction, and life history, affecting their adaptability, behavior, and distribution. In China, *B. tau* may be affected by the short-term high-temperature periods that have become increasingly common during the summer, causing changes in their growth and metabolism. In order to closely simulate the external temperature effects, we selected a range of treatment temperatures to treat *B. tau* in the laboratory. Our results showed that short-term high-temperatures do have a significant effect on the growth and reproduction of different stages of the fly.

Increasing temperatures beyond a given threshold are detrimental to the development of insects. Excessive temperature are harmful if not lethal to most insects^40–42^. When exposed to the higher temperature treatments between 34 °C to 48 °C, the corrected survival rate of each developmental stage of *B. tau* gradually decreased with each incremental increase of temperature. After 12 hours of treatment, the LC_50_ of *B. tau* at different high-temperatures was the highest in pupae, lowest in eggs, and slightly higher in adult females than in adult males [pupae (42.06 °C)> adult females (40.447 °C)> adult males (40.013 °C)> eggs (38.31 °C)> larvae (36.74 °C)]. In other words, at high-temperatures, pupae were able to survive the longest, while the eggs were the most susceptible of the stages, and adult females were slightly more resistant to the heat than adult males. The high heat tolerance seen in the pupae may be explained by the Bogert effect^43^. According to this principle, the different stages of insects can be divided into mobile and non-mobile stages. The adult and larva are mobile stages, and the eggs and pupae are non-mobile stages. Insect are capable of changing their behavior as well as their physiological metabolism to avoid the damage caused by high-temperature stress when the ambient temperature rises. Insects that are in the mobile stage (adults and larvae), may have the potential to escape their current detrimental environment and avoid high-temperature damage. However, insects that are in non-mobile stages (eggs and pupae) in similar situations can withstand the adverse effects only by improving their innate heat resistance. As a result, the heat resistance of non-mobile insect stages is normally higher than it is during their mobile stages^44,45^. In *Drosophila buzzatii* (Patterson & Wheeler) and *Drosophila suzukii* (Matsumura) (both Diptera: Drosophilidae) the pupae have been shown to be the most heat resistant stage, and have a wider temperatures range for survival than the other life stages^46,47^. Therefore, short-term high-temperatures do have a significant effect on every developmental stage of the fruit flies. These results are similar to those of other studies. For instance, Ma *et al*. (2010) noted that the longevity and fecundity of the aphid *Metopolophium dirhodum* (Walker) (Hemiptera: Aphididae) were significantly decreased when the temperature was increased from 29 °C to 34 °C^48^. The survival pattern, longevity, and fecundity of *Corythucha ciliata* (Say) (Hemiptera: Tingidae)were reduced when they were heat shocked at 43 °C and 45 °C^49^; It has also been reported that survival rate decreased in the whiteflies *Trialeurodes vaporariorum* (Westwood) (Hemiptera: Aleyrodidae) and *Bemisia tabaci* (Gennadius) (Hemiptera: Aleyrodidae) B-biotype as temperatures rose^50^. Another study demonstrated that the locust *Calliptamus italicus* (L.) (Orthoptera: Acrididae) was able to survive higher temperatures by regulating the contents of stress-resistant substances and a protective enzyme at certain temperature ranges, but due to differences in the contents of the stress-resistant substances and the protective enzyme and it was shown in a growth rate comparison that the females were capable of a faster response rate and had a greater high-temperature resistance^51^.

Temperature affects the growth and reproduction of insects. Within a given range, the growth rate of insects will increase incrementally corresponding to gradual temperature increases, but as the damaging period is prolonged, the amount of damage inflicted can increase algebraically^52^. If the temperature continues to rise, it becomes detrimental to the development of the insect, with excessive temperatures adversely affecting their development^53–55^. The developmental duration of *B. tau* was prolonged as the high-temperature treatment was increased from 34 °C to 42 °C. The growth periods of the egg, larval, and pupal stages of *B. tau* were significantly extended over that of the control temperature when they were stressed at 42 °C. Within the above range of higher temperature then, the developmental period increases with increasing temperature. Studies have reported that in the suitable temperature range, the length of the immature stage will gradually shorten as the temperature increases^56^. Conversely, the developmental duration of the insect was prolonged when the ambient temperature was elevated to an unsuitable temperature. Similar results were found in other stresses; for instance, increased atmospheric CO_2_ concentration significantly prolonged the development period of *Spodoptera frugiperd*a (J. E. Smith) (Lepidoptera: Noctuidae)^57^. In *B. tau* the ideal developmental temperature regime was found to be relatively narrow and at temperatures less than 34 °C. Because temperature ultimately determines the geographical distribution^58,59^, phenology^60^, and population richness^61,62^by affecting the growth and development rate, behavior, survival, and reproduction of a species, it is important that we identify the effects that predicted higher temperature will have on insects, particularly pest species.

In the natural environment insects are often stressed by adverse factors. Of these, temperature is one of the most important factors because insects are directly affected by this variable-considerably more so than their response to man’s attempts to control them. In their long-term survival strategy, insects possess the adaptability and evasion ability to adapt to a multitude of external factors. In the present study, when temperatures were increased from 24 °C to 42 °C, the longevity of adults initially increased and then decreased, with females surviving longer than the males. The female egg production showed a downward trend as temperatures were raised from 24 °C to 40 °C. Adult longevity were gradually prolonged as temperatures increased. After treatment at different temperatures from 24 °C to 42 °C, the ratio of the female offspring initially increased and then decreased, attaining a maximum at 38 °C, and then exhibited a gradual decline in production. When the ambient temperature exceeded 34 °C, the developmental period of *B.tau* was prolonged and the reproduction of *B.tau* was reduced^34^. It is extremely unlikely that *B. tau* would be capable of survival as a viable species at tempertures in excess of 40 °C based on the offspring exhibiting a distinct downward trend at this temperature. When treatment temperatures were between 24 °C and 38 °C, *B. tau* showed a trend favoring females in the ratio of female/male offspring, producing an excess of females which would allow the reproduction rate to increase in order to maintain or increase the size of the population. When an insect experiences adverse temperature stimuli, it can resist the high and low-temperature threats by synthesizing stress-resistant proteins or other metabolites^63^. These substances can reduce the damage caused by the unfavorable environment to insect growth and may have corresponding compensation effects, i.e., the increase in longevity of insects after experiencing injurious temperature stimulation may be related to compensation^64^. However, the synthesis of these substances requires the addition of additional substances and energy. If the resources and energy are not supplemented, the life of the insects may be shortened. In this study, the adult lifespan increased after treatment at a temperature of 34 °C, but at the expense of fertility.

In summary, short-term high-temperature stress adversely affects the survival rate, developmental duration and adult longevity, single female egg production, and the female ratio of *B. tau* offspring.

## Conclusion

Short-term high temperatures over 42 °C are not suitable for the growth and development of *B. tau*. A short-term high-temperature of 40 °C is not suitable for successful reproduction in *B. tau*. Therefore, in programs aimed at prevention and control of the insects during the warmer weather season, attention should be concentrated on the number of visible oviposition sites to guide the timing of pesticide applications. At the quarantine port, when *B. tau* infestations are subjected to short-time high-temperature, the temperature should exceed 48 °C for 12 hours to insure that all *B. tau* have been killed.
